# Circles reshaping the RNA world: from waste to treasure

**DOI:** 10.1186/s12943-017-0630-y

**Published:** 2017-03-09

**Authors:** Jing Liu, Tian Liu, Xiaman Wang, Aili He

**Affiliations:** Department of Clinical Hematology, Second Affiliated Hospital, Xi’an Jiaotong University Health Care Center, 157 West 5 Street, Xi’an, 710004 Shaanxi People’s Republic of China

**Keywords:** Circular RNA, miRNA, Biomarker, Post-transcription regulation, Cancer

## Abstract

A new type of RNAs was identified from genes traditionally thought to express messenger or linear ncRNA (noncoding RNA) only. They were subsequently named as circRNAs (circular RNAs) due to the covalently closed structure. Accumulating studies were performed to explore the expression profile of circRNAs in different cell types and diseases, the outcomes totally changed our view of ncRNAs, which was thought to be junk by-products in the process of gene transcription, and enriched our poor understanding of its underlying functions. The expression profile of circRNAs is tissue-specific and alters across various stages of cell differentiation. The biological function of circRNAs is multi-faceted, involving five main features (sponge effect, post-transcriptional regulation, rolling circle translation, circRNA-derived pseudogenes and splicing interference) and varying differently from the locations, binding sites and acting modes of circRNAs. The regulating role of circRNAs is not isolated but through an enormous complicated network involving mRNAs, miRNAs and proteins. Although most of the potential functions still remain unclear, circRNAs have been proved to be ubiquitous and critical in regulating cellular processes and diseases, especially in cancers, from the laboratory to the clinic. Herein, we review circRNAs’ classification, biogenesis and metabolism, their well-studied and anticipated functions, the current understanding of the potential implications of circRNAs in tumorigenesis and cancer targeted therapy.

## Background

CircRNA is an up-rising star in ceRNAs (competing endogenous RNAs) field, even in the whole RNA world, characterized by the distinct structure without terminated 5’ caps and 3’ tails [1]. Although it was previously discovered as errors in RNA splicing process, its unique properties and powerful functions are being increasingly acknowledged by scientists. Natural circRNA has been proved as an abundant, stable, diverse and conserved class of RNA molecules which plays an indispensable role in the RNA interaction network [2]. CircRNA was found extensive in eukaryotic cells by deep sequencing technologies, and a large number of researches suggested that it possesses complicated functions such as competing with linear RNAs in the splicing, working as endogenous RNAs to sponge miRNAs, regulating transcription and so on [3, 4], and it seems that other important but underlying functions will be revealed as soon as possible. Moreover, circRNA was confirmed to be associated with varied cellular process and involve in the biogenesis and development of many diseases, especially in cancer [5–8], its potential of serving as diagnostic or predictive biomarkers of disease has become a hotspot in scientific field up to now. In this review, we describe the biogenesis, classification, functions and potential implications in cancer targeted therapy of circRNA, telling the story of how circRNA switches from “nobody” to “hero”.

## Main text

### The discovery and distribution of circRNA: a beautiful accident with dramatic impact

CircRNAs are a group of non-coding RNAs that are ancient yet newly recognized regulating molecules. After the earliest discover in plant-based virus in 1976 [9], they just come into scene in recent years and are found widely spread in eukaryotic cells from flies to mammals and humans thanks to the technological breakthroughs in high-throughput deep sequencing [10–12]. But still, circRNA was regarded as the functionless junk-RNA developed from mRNA splicing thus did not get enough attention as they should be. Until 2013, a famous and classic paper published by Hansen TB et al. on the journal of nature has totally changed the fate of circRNA and turn it into a focal point of scientific research [3]. The subsequent findings indicate that circRNAs have great potential to perform special regulating roles in biological development. Thousands of circRNAs were also suggested to present in plant cells and might act as a negative regulator of its parental gene through genome-wide identification and potential function analysis of circRNAs in rice [13]. Similar with lncRNAs, the expression profile of circRNAs is specific among different cell types indicating its possible regulatory function. More and more studies have suggested that they contribute disease initiation and progression, possess the potential of turning into new clinical diagnostic and prognostic markers, and serve new insights into the therapy of cancers.

### The formation of circRNA: a slow but ingenious process

Although more and more human circRNAs were identified and uploaded online using molecular biology strategies combined with new bioinformatic approaches, the underlying mechanisms of circRNA generation are still not clear yet. There is an assumption that at least four distinct paths involved in the formation of circRNAs [2, 10, 14, 15].

1. Spliceosome-Dependent Circulation Path.
  Most eukaryotic circRNAs are generated during alternative splicing, an essential step in the eukaryotic gene expression process that is catalyzed by either spliceosomal machinery or group I/II ribozymes [16–18]. The processing of the back-splicing reaction seems to require the canonical spliceosome, a kind of cis-regulatory elements which are able to regulate splicing and catalyse circulation [12, 19]. Although the detailed mechanism about how the spliceosome is involved in back-splicing remains unknown exactly, it is reasonable to propose that following the sequential assembly of snRNPs (spliceosomal small nuclear ribonucleoproteins) on the pre-mRNA, the downstream 5′ donor site of an exon is combined to an upstream 3′ acceptor site to catalyse circRNA biogenesis. We already knew that the back-splicing efficiency is very low compared to the canonical splicing, this is possibly due to the fact that spliceosomes are unfavourably assembled at back-splicing.
2. Intron-Pairing-Driven Circularization Path and Lariat-Driven Circularization Path
  Firstly, in the ‘Intron-Pairing-Driven Circularization Path’ (Fig. 1b), a large number of circRNA are formed depend on this intronic reverse complementary motifs, which contain a seven-nucleotide GU-rich element close to the 5’ splice site and an 11-nucleotide C-rich element near the branch point site [15, 20]. The donor-acceptor pairs on the primitive transcripts can be brought close enough to promote the circularization of exons through alternative 5’ to 3’ splicing [14, 21, 22]. Secondly, in the ‘Lariat-Driven Circularization Path’ (Fig. 1b), the classical skipping process leads to the generation of the lariat precursor, which is by-products in exon-skipping event happened during canonical linear alternative splicing [2], but represents a key attribute of this model. The circularization of extros and intros can be promoted by this specific lariat structure (Fig. 1c). In these two classical models of circularization, ALU complementary flanking elements (retrotransposons characterized by the action of the Arthrobacter luteus (Alu) restriction endonucleas) repeated in intronic regions compete with canonical linear-RNA splicing and act as a necessary accelerator in the formation of circRNAs by reverse complementary matches [2, 12, 14]. IRAlu (reverse Alu element) has already become an important basis of analyzing and forecasting the formation mechanism of circRNA [23].

**Fig. 1:**
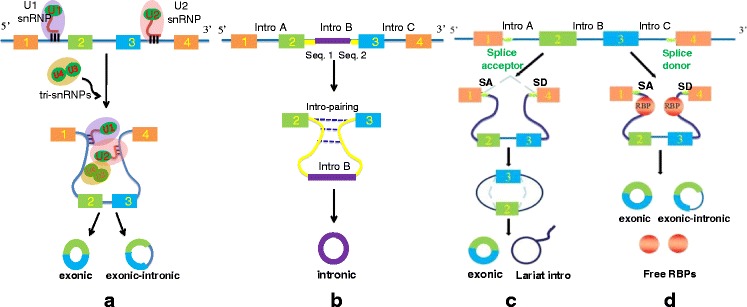
Three paths of circRNAs formation. Legend: There are four commonly acknowledged paths in the formation of circRNAs. **a** Spliceosome-Dependent Circulation Path: the processing of the back-splicing reaction requires the sequential assembly of snRNPs on the pre-mRNA to catalyse circulation. **b** Intron-Pairing-Driven Circularization Path: circRNA are formed depend on an intronic reverse complementary motif containing a GU-rich element and a C-rich element in two sides to promote circularization. **c** Lariat-Driven Circularization Path: formation of circRNA is promoted by a lariat structure; the ALU complementary flanking elements repeated in intronic regions compete with canonical linear-RNA splicing and accelerate the circularization by reverse complementary matches. **d** Protein Factors Associated Circulation Path: some RBPs can bind to specific targets in introns and bring the donor-acceptor sequences in proximity to each other thus trigger several circularization processes. At the mean time, RBPs also stabilize the splicing motifs and inhibit canonical linear splicing
3. Protein Factors Associated Circulation Path
  As in the ‘Intron-Driven Circulation Path’, some RBPs (RNA binding proteins) can bind to specific targets in introns and bring the donor-acceptor sequences in proximity to each other thus trigger several circularization processes instead of the intronic reverse complementary motifs. In this model of circularization, RBPs play a double role of both stabilizing the splicing motifs and inhibiting canonical linear splicing in the process of regulating circRNA generation (Fig. 1c). QKI, one of the well studied RBPs, involve in the process of human EMT (epithelial-mesenchymal transition) by regulating circRNA generation thus play a role in the neoplasm metastasis. Insertion of QKI binding sites into linear RNA can induce exon circularization [24]. Besides the positive functions mentioned above, some RBPs play a negative role in the process of circRNA formation. For instance, ADARs (Adenosine deaminases acting on RNA) were suggested to inhibit circRNA formation by melting the stem structure [14]. ADARs are highly conserved proteases binding with double-stranded RNA, mainly involve in RNA editing, particularly the process of transforming the adenosine residues in double-stranded RNA into creatinine [25]. Compared with the host mRNA, expression level of circRNA was observed an increase after ADAR knockdown, which indicating the negative regulating role of ADAR in the circularization of RNAs [14].
4. Foramtion rate is actully slow: Long time accumulation makes the abundance
  Previous researches of circRNAs are all reported in genomics level, since the relatively stable characteristics of circRNA can achieve its accumulation within a certain time. The rate of circRNA formation is still a mystery until now. A newly published paper showed that the forming rate of circRNA is actually very low, and the generation process is related to the rapid extension of the polymerase II. The researcher took the neurons as example and drew a final conclusion that circRNA depends on the cis-elements (reverse complement sequence inside RNA) in the process of rapid transcription and the long-time cumulative effect in cells [26]. We have to admit that the mechanism of circRNA biogenesis is so complicated and related to varied regulatory factors. The definitive explanation of their interaction during the formation of circRNA still needs further research.

### Classification of circRNA: a complicated system possessing its own special rhythms

Due to the confusion of nomenclatural method in different databases and the poor understanding in biogenesis and characteristics of circRNAs, generating a systematic circRNAs classification is not an easy task. Up to now, there are two separate ways to divide circRNAs based on different principles.

1. Three subsets classification: component-oriented
  According to the most existing studies, comprehensive circRNAs can be classified into three types (Fig. 2), exonic circRNAs, intronic circRNAs, and exon-intron circRNAs, focusing on their components [27]. Just as its name implies, ecircRNAs (exonic circRNAs) comprise exons generated in the process of exon skipping in pre-mRNA sequences, and they are likely to be transported to cytoplasm by the nuclear export system, or escape from nuclei during cell division [28]. Cytosolic localization of circRNAs may also support their post-transcriptional function. CiRNA (intronic circRNAs) is composed by two or more connected introns and was detected rare in eukaryotic cells. EIciRNAs (Exon-intron circRNAs) are circularized with introns ‘retained’ between the exons, they could be overexpressed with their flanking complementary sequences. Both ciRNAs and eIciRNAs position on transcription sites in nucleus and have the potential function of promoting the transcription of their parent mRNAs [29, 30].

**Fig. 2:**
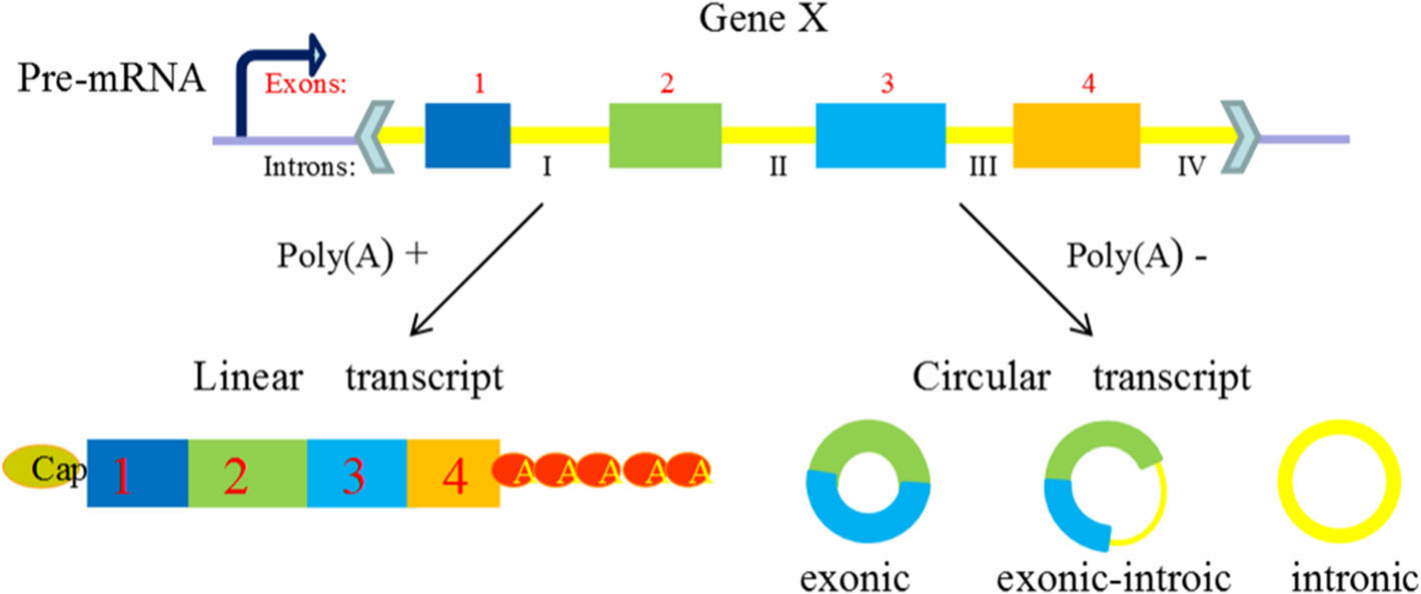
Composition of three types circRNAs. Legend: CircRNAs are generated in the process of the skip splicing of pre-mRNA and compete with the linear transcription, they are generally classified into three types focusing on their components. (1) EcircRNAs comprise exons and are the most common circRNAs. (2) CiRNAs are composed by two or more connected introns and are detected rare in eukaryocyte. (3) EIciRNAs are circularized with introns ‘retained’ between the exons and play a part in gene regulation
2. Five subsets classification: location-oriented
  With the development of deep sequencing in varied species and tissues, some special circRNAs types was discovered, circRNAs should be classified into five types, “exonic”, “intronic”, “antisense”, “sense overlapping” and “intergenic” according to their location relationship with adjacent coding RNA (Fig. 3). Except for those two common types composed of intros and extros, “antisense” represents circRNA whose gene locus overlap with the linear RNA, but transcribed from the opposite strand; “sense overlapping” represents circRNA transcribed from same gene locus as the linear transcript, but not classified into “exonic” and “intronic”; “intergenic” represents circRNA located outside known gene locus. This way of classification focusing on the location of circRNA in prime transcripts is more beneficial to research the interaction between circRNA and their adjacent coding RNA in some degree.

**Fig. 3:**
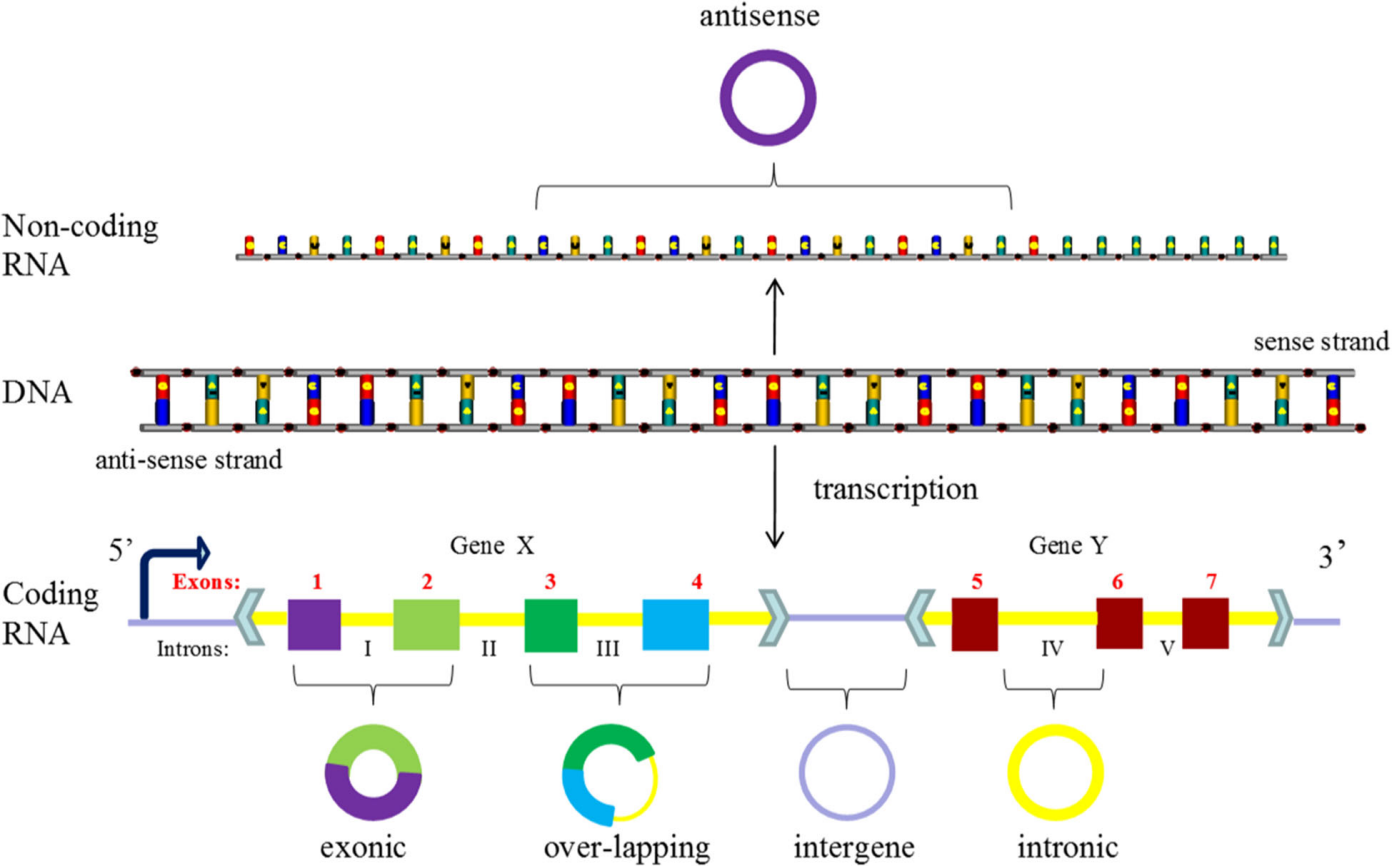
The new five subsets classification of circRNAs. Legend: CircRNAs can be divided into five groups according to their location relationship with coding RNA. “exonic” and “intronic” represent circRNA composed by exons and intros respectively, “antisense” represents circRNA whose gene locus overlap with the linear RNA, but transcribed from the opposite strand; “sense overlapping” represents circRNA transcribed from same gene locus as the linear transcript, but not classified into “exonic” and “intronic”; “intergenic” represents circRNA located outside known gene locus

### Useful tools in circRNA research: complementation of colors makes the whole spring

Large-scale RNAseq has been widely employed for many purposes in scientific research and substantially changed the transcriptomics field, especially the circRNAs, as it enables an unprecedented amount of high resolution data to be acquired. Except for the established softwares which are powerful enough to analyze the sequence data, several current online databases containing circRNAs collected from GenBank annotations or published articles enable researchers to organize the circRNAs conveniently.

1. Online-databases of circRNA: different flowers bloom together
  There are many free online databases available for the circRNA research, each database has its own characteristics and covers the different part of the information about circRNAs. For instance, both starBase v2.0 (http://starbase.sysu.edu.cn/ Accessed 10 Augst 2016) and circBase (http://www.circbase.org/ Accessed 10 August 2016) allow the user to search for functional classes or processes [31, 32]; Two disease-oriented database, namely circ2Traits (http://gyanxet-beta.com/circdb Accessed 21 July 2016) and nc2Cancer (http://www.Bioinfo.Tsinghua.Edu.cn/nc2Cance Accessed 15 July 2016), provide plenty of information about cirRNA-associated diseases and the genomic positions of several circRNAs [4, 33]. While CircNet (http://circnet.mbc.nctu.edu.tw/ Accessed 12 September 2016) and deepBase v2.0 (http://biocenter.sysu.edu.cn/deepBase/ Accessed 23 August 2016) contains the most CircRNA annotation (Annotation of circRNAs depends on sequencing reads spanning the backsplice junction and thus map as nonlinear reads in the genome), especially human CircRNAs [34, 35]. CircInteractome (http://circinteractome.nia.nih.gov Accessed 8 September 2016) will be the only option if you want to match the circRNA with relevant RNA-binding proteins [36]. As for the search for sequence alignment of circRNAs, circBase and CircNet would be the perfect choice because they are equipped with a web interface of BLAST. And CircNet is the only one to provide circRNA expression profiles in samples. There is no doubt that every database present with its own sphere of competence, only the perfect combination of various database can provide information in a reliable way.
2. A newly established tools with pipeline analysis of ceRNAs.
  Except for the online database, the vast majority of the existing softwares are very valuable and efficient in analyzing the raw data from new generation sequencing, while most of them still pose a challenge to the researchers without basic programming and computing skills. Most importantly, the circRNAs outputs from five different algorithms: circRNA_finder [37], find_circ [38], CIRCexplorer [23], CIRI [39], and MapSplice [40], are observed surprisingly dramatic differences, specifically regarding the highly expressed circRNAs and the circRNAs derived from proximal splice sites [41]. In order to bring together these well-established tools in a single bundle and achieve reliable predictions, Andrés-León, E. et al. developed a suite named miARma-Seq [42]. This is a comprehensive pipeline analysis which can identify mRNAs, miRNAs and circRNAs in any sequenced organism and enable differential expression, miRNA-mRNA target prediction or functional analysis. It runs in three different operative systems and has been tested for reliance on software updates, demonstrating the utility of this tool for the research community [42].

### Conservation of circRNAs among species

As a “bridge” between DNA and protein, the complex regulatory role of RNA has long been underestimated. In eukaryotic cells, protein-coding RNA (mRNA) only occupies about 2% of the genome, the rest massive number of transcripts were classified to non-protein coding RNAs (ncRNAs). CircRNA was found stable and abundant in cells across species, involving in complex regulatory networks of ncRNAs and playing a indispensable role in the development of non-neoplastic diseases and tumors [43, 44].

1. Assumption about circRNA/linRNA ratio across tissues
  Why would circular RNAs get so much attention? Recent papers have described the presence of circular RNA species and their linear counterparts in different tissues, the outcomes showed that the ratio of circular to linear RNA isoforms was always low in colon tumor compared to adjacent normal colon tissues and even lower in colorectal cancer cell lines. This phenomenon also presents in ovarian cancer and its adjacent normal samples [45]. Furthermore, circRNA/linRNA ratio correlates negatively with the proliferation index of cells. This negative correlation seems to be a general principle in human tissues and different kinds of cell lines. There is a chance that circular and its counterpart linear RNAs are synthesized by specific splice activities in a gene (cell type and condition) dependent ratio. As for proliferating cells, if both RNA species are evenly distributed to offspring cells during proliferation, linear RNAs are accurately controlled by active transcription and degradation also leading to newly expressed circRNAs, while circular RNAs are much more stable and degradation-resistant. This may be one of the mechanisms to maintain the constant ratios of circular to linear isoforms. In contrast, in non-proliferating cells stable circular RNAs accumulate whereas linear transcripts are in a regulated steady state of transcription and degradation. This accumulating assumption explains perfectly why the global circRNA abundance of brain is much higher than other human tissues.
2. Serum exosomes: an efficient circRNA carrier in human body
  Serum exosomes are a kind of membrane vesicles secreted by maternal cells, containing a bunch of proteins, mRNAs and miRNAs, possessing varied functions such as transmitting the biological information, regulating the behavior of recipient cell and so on [46, 47]. The latest study found that there are more than 1000 kinds of circRNAs in exosomes, wherein the enrichment degree of circRNAs (exo-circRNA) is even greater than the mother cell secreting the vesicles, circRNA/linRNA ratio in exosomes is also 6–10 times of the mother cell [48]. This conclusion has been extensively validated in lung, colorectal, breast, stomach, liver and cervical cancer cell lines [49]. Exo-circRNAs resist degradation of RNA exonuclease and maitain stablity after 24 h’ incubation in serum at room temperature. They can eliminate the inhibition effect mediated by associated miRNAs in recipient cell. Thus exo-circRNA has been proved to exist stably in serum and still retains the original ring like structure and biological function. Also, its abundence is closely related to the extent of the tumor load [48].

### Powerful functions of circRNA: from the waste to treasure

**Fig. 4:**
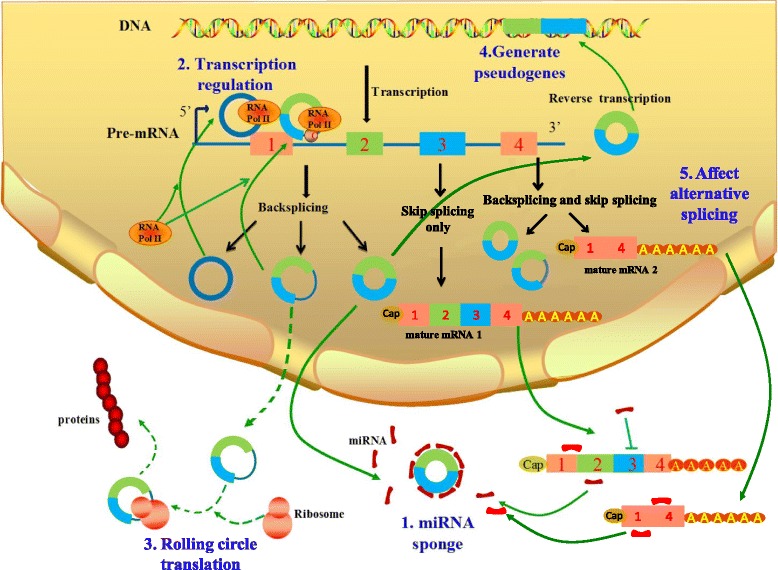
Five main functions of circRNA. Legend: CircRNAs possess five main functions which have been validated in some related researches. 1. MiRNA sponge: circRNAs serve as the platform to bind miRNAs and affect their biological function, regulating the activity of miRNA-target gene. 2. Post-transcription regulation: the stable ciRNA and EIciRNA locate in nucleus, binding to elongating RNA Pol II and promoting transcription. 3. Rolling Circle Translation: some circRNAs can translate into proteins via a rolling circle amplification mechanism, while only the vitro circRNA till date has been verified to encode proteins in eukaryotic cells. 4. Generating the circRNA-derived pseudogenes: some circRNAs may be reversely transcribed to cDNA and integrated into the genome based on an unknown way. 5. Affects alternative splicing: circRNA biogenesis can compete with pre-mRNA splicing, resulting in lower levels of linear mRNAs and changing the composition of processed mRNA by excluding specific exons from the pre-mRNA

Just several years ago, circRNA was still considered the waste of error splice, while up to now, they were proved gradually to possess better stability, escape the degradation of RNAase and widely spread in eukaryotic cells [1, 49, 50]. These deeper understanding of circRNAs’ characteristics paves the way for the reveal of their four crucial functions (Fig. 4).

1. A super sponge for miRNA
  CircRNAs has been identified as a new kind of ceRNAs that sponge and inhibit relevant miRNAs by complementary base paring (Fig. 4.1). MiRNA was believed to bind to the complementary site on the 3′ UTR (untranslated region) of targeting mRNAs, inhibiting protein translation or promoting mRNA degradation on a post-transcriptional level [51, 52]. MiRNAs are closely related to various ways of regulation, including body development, virus defense, hematopoiesis, organ formation, cell proliferation and apoptosis, fat metabolism, and so on [53–56]. They have also been reported to act as potential cancer biomarkers [56].
  CircRNAs can affect the biological function of miRNA, regulating the activity of miRNA-target gene, thus playing an indirect role in the regulation on post-transcriptional level. CiRS-7 is a recently identified human circRNA which harbors more than 70 conventional miR-7 binding sites thus works as a super-sponge of miR-7 [3, 38]. It was observed overlapping co-expression with miR-7 in the mouse brain, particularly in neocortical and hippocampal neurons. It is resistant to miRNA-mediated target destabilization, which is normally facilitated by exonucleolytic decay, thus effectively suppresses miR-7 activity and results in increased levels of miR-7-targeted transcripts [38]. Another circular RNA called SRY, contains many binding sites for miR-138 and functions as a miR-138 sponge [3, 57]. CirRNAs’ strengthen of miRNA-binding capacity is much powerful than that of any other ceRNAs [58], it is why they are always call the “super sponge”. The self-cleavage of circRNAs can lead to the release of a large number of inactive miRNA;
2. Post-transcription regulation
  Most ciRNA (formed by the introns) position in the nucleus, almost has no miRNA binding function, yet what is intriguing is that knocking down ciRNA will hinder the transcription of the corresponding gene [21]. Ci-ankrd52 substantially enriches in transcription initiation region of the relevant gene, and promotes the function of RNA polymerase II. This study preliminarily proved that the function of ciRNA is promoting transcription of corresponding gene. EIciRNAs (formed by exon and intro) also predominantly localize in the nucleus and serve as a regulator for transcriptional control. They were revealed not only interacting with U1 snRNP but also enhancing the expression of their parental genes by specific RNA-RNA interaction between U1 snRNA and EIciRNAs [29].
  CircRNA can work together with RBPs to form a complex, inhibiting transcription, or directly regulating the expression of mRNA through Watson-Crick base pairing rules [38] (Fig. 4.2). Stable circRNAs with elongated RNA Pol II binding sites have the function of mediating alternative splicing and transcription by binding to RNA Polymerase II [21]. Except for QKI, RBP MBL (muscleblind) is another protein which has been shown to bind to flanking introns of linear transcript and trigger the generation of circMbl (circular muscleblind) [12]. The functional defects of MBL is known to cause a serious degenerative disease called myodystrophy-derived myotonia [59], which is the most common form of muscular dystrophy of adulthood characterizing by progressive muscle atrophy and weakness. On the other hand, circMbl can strongly compete with canonical splicing and regulate MBL levels through the highly conserved MBL-specific binding motifs within the sequence of circMbl [12]. We can guess that circRNAs may have a strong association with the understanding and ultimate treatment of muscle degenerative diseases.
  There are also many other circRNAs containing translation-initiating site possess the similar function and act as mRNA trap to regulate expression of their host gene. It hence appears that involving in the regulation of gene transcription process is another important functional model of circRNA except for miRNA Sponge.
3. Rolling Circle Translation
  Circular mRNA have been proved to be translated in eukaryotic cell if it contains a IRES (internal ribosome entry site) sequence combining directly with ribosome [60–62]. The eukaryotic ribosomal 40S subunits can initiate the translation both in vitro and in vivo once they bind to circRNA at the entry point [63–65]. Furthermore, Naoko Abe, etc. found that circular RNA containing an ORF (infinite open reading frame) can also be efficiently translated to produce proteins in an Escherichia coli cell-free translation system [62]. There are also other evidences showing that circRNA inserted with a GFP (green fluorescent protein) open reading frame can translate GFP successfully in E.coli [63, 66]. What’s maybe more interesting is that some circRNAs can be translated in living human cells independent of the participation of the third parts like internal ribosome entry site, a poly-A tail, or a cap structure, which is essential for internal initiation in conventional translation. Moreover, proteins could be synthesised from these circular RNAs in eukaryotic translation systems via a manner similar to rolling circle amplification (RCA) of the polymerase reaction (Fig. 4.3). Without the need to bind multiple times with the RNA template, circRNAs can not only produce a long-repeating peptide sequence, but also enhance the productivity over a given period of time compared to its linear counterpart [62]. These findings told us that translation of exonic circRNAs present in human cells is more probable than previously thought. However, only the vitro circRNA is known to encode proteins in eukaryotic cells, there is no direct evidence till date to prove that natural eukaryotic endogenous circRNAs can be translated.
4. Generating the circRNA-derived pseudogenes
  Given the unique structure, highly conservative sequence, absolute stability and rich content of circRNAs, is there any chance that stable circRNA molecules could possibly be reversely transcribed and integrated into the genome, forming the RNA-derived pseudogenes? Researchers analyzed the corresponding circularization sites (exon 6- exon 2) of circRFWD2 in mouse genome, found 33 “highly credible circRFWD2-derived pseudogene”, 9 “lowly credible circRFWD2-derived pseudogenes “, and six pseudogene containing exon sequences located outside of circRFWD2. LINE-1 mediated RNA reverse transcription usually require the existence of Poly (A), yet 39 of 42 circRFWD2 related pseudogenes contain no Poly (A) sequence, This illustrates that some circRFWD2 may be reversely transcribed to cDNA and integrated into the genome based on an unknown way (Fig. 4.4).
  In addition circRFWD2, there are other cases of circRNA-derived pseudogene, such as circSATB1-derived pseudogenes presenting in the genome of all known mouse strains and circDIAP3-derived pseudogenes only presenting in parts of the strains. The circPRKDC and circCAMSAP1-derived pseudogenes found in human genome also present in gorillas and chimpanzees, but do not exist in rhesus monkeys. Interestingly, the circSATB1-derived pseudogene discovered in mouse MEL and G1E cell lines contains CTCF binding sites, which are missing in exon region of corresponding SATB1 gene. The human genome shows no a similar situation.
  This strange phenomenon reminds us that we should be careful about the interference of circRNA-derived pseudogenes on the experimental results during the study circRNA. Although the total quantity is low up to now, the finding of circRNA-derived pseudogenes hints a previously regardless impact of circRNAs on host genome by retrotransposition.
5. Cyclization process affects alternative splicing
  CircRNA is generally accepted as a unique functional by-products derived from splicing process of protein-coding genes. A large proportion of circRNAs we identified till date are exon-derived. Thus, the forming process of such circRNAs could affect alternative splicing of the relevant pre-mRNAs, potentially leading to altered gene expression (Fig. 4.5). A paper published in Circulation Research pointed that RBM20 is crucial for the formation of a subset of circRNAs that originate from the I-band of the Titin gene. In RBM20-knockout mice, exons that originally corresponded to the formation of circular RNA were found more concentrated in linear RNA [67]. The authors speculate that these exons may be part of the precursor RNA transcribed by Titin gene, they are cleaved from precursor RNA molecule and further forms circRNAs with the aid of RBM20 in a normal situation. When RBM20 is mutated or knocked out, these exon sequences cannot be normally cut down to start the cyclization, and finally accumulate in the linear RNA molecules.
  On the contrary, circRNA biogenesis can compete with pre-mRNA splicing, resulting in lower levels of linear mRNAs and changing the composition of processed mRNA by excluding specific exons from the pre-mRNA. Although no natural eukaryotic endogenous circRNA is yet proved to possess the function of translating into proteins, considering the principle we mention above that the more exons are circularized, the less they will be present in the processed mRNA, it is reasonable to speculate that the overexpression or deletion of some gene splicing associated proteins may also modulate formation of mRNA by affecting the amount of circRNAs, further regulate gene expression. This theory will offer a brand-new perspective for the nosogenesis research of some disease.
  In consideration of the numerous unsolved problems about circRNAs as yet, more reliable researches are needed to discover the potential existence of other unknown circRNAs or functional RNAs, and complete their functional characterization in eukaryotic cells.

## Conclusion

### CircRNA in CNS: a burgeoning aging milestone

The enormous abundance of circRNAs in brain compared to other normal or cancerous human tissues announces their preferred derivation from neural genes and intensive accumulation in neural tissues [68]. But this dose not cover the whole story, circRNAS was revealed increasing substantially relative to their linear isoforms during CNS aging. This age-related accumulation of circular RNA might have implications for its potential of working as a promising aging marker [37]. Besides the apparent aging indicators like skin winkles and gray hair, circRNA might be able to tip the aging of brain and internal organs in advance, alarming human to take some preventive measures before it is too late. In addition, many brain circRNAs changed their abundance abruptly at a time corresponding to synaptogenesis, they also exhibit a substantial up- or downregulation when the homeostasis of neuronal activity changes [19]. Brain circRNAs are suggested to regulate synaptic function and go hand in hand with the development of nervous system, this important discovery adds further dimension to our understanding of the molecular pervasiveness and importance of circular RNAs.

### CircRNAs in non-neoplastic diseases: a double-edged sword

CiRS-7 was firstly found in large amounts in human brain tissue and was also termed CDR1 (cerebellum degeneration-related antigen 1, CDR1,also known as CDR34) because of its transcription in the antisense orientation with respect to the CDR1 gene, which has been implicated to be crucial in autoimmune neurologic disorders like Alzheimer’s disease [69–71]. CircRNA CANRIL was identified to be relevant to the risk of human atherosclerosis [2]. Also, the over-expressed HRCR (heart-related circRNA) attenuates the development of cardiac hypertrophy and heart failure in mice by directly binding to miR-223 and acting as an endogenous super sponge to inhibit miR-223 activity, which results in the increasing expression of ARC, a target for miR-223, in heart cells [6].

Some circRNAs are even able to possess the function of regulating cell cycle progression. Circ-Foxo3 is a highly expressed circular RNA in non-cancer cells, silencing endogenous circ-Foxo3 accelerates cell proliferation while ectopic expression of circFoxo3 hampers cell cycle progression [72]. Circ-Foxo3 was shown to bind with the cell cycle proteins CDK2 and p21 to form a ternary complex. Normally, CDK2 (cyclin-dependent kinase 2) is a well-known cell cycle facilitator interacting with cyclinA and cyclinE [73], while p21 (cyclin-dependent kinase inhibitor 1) restrains these activities and arrest cell cycle progression [74]. The formation of circ-Foxo3-p21-CDK2 ternary complex blocks cell cycle progression by arresting the function of CDK2 [72]. May be the dysregulation of circ-Foxo3 is relevant to the neuromuscular degenerative diseases or even the germination of cancer.

### CircRNAs in cancer: a promising biomarker

Some researchers provide evidences that circ_001569 acts mechanically as a miRNA sponge to inhibit miR-145 activity, and subsequently up-regulates miR-145 targets E2F5, BAG4 and FMNL2 to promote cell proliferation and invasion in CRC (colorectal cancer) [7]. Cir-ITCH was also shown to inhibit the development and progression of CRC and ESCC (esophageal squamous cell carcinoma) [75, 76]. Nowadays, a larger number of circRNAs was found to significantly express differentially between ovarian epithelial carcinoma and normal adjacent tissue than mRNA. Furthermore, the expression profile of circRNA in primary ovarian epithelial carcinoma and metastatic carcinoma is also different [8]. Hsa_circ_002059 has been proved to be down-expressed in gastric tumor samples compared to the normal gastric mucosa and may be used as a potential biomarker in the early diagnosis of gastric cancer [77]. The hsa_circRNA_104912 level was significantly lower in LSCC (laryngeal squamous cell carcinoma) than in corresponding adjacent non-neoplastic tissues, playing an important role in the tumorigenesis of LSCC [78]. We can assume that perhaps the different expression of circRNA is associated with tumor metastasis and invasion. In brief, circRNAs are gradually found to play a potential and necessary role in the biogenesis of all kinds of tumors. Further, circRNAs present a more robust expression pattern across patients than their linear counterparts indicating their suitability as biomarkers in highly heterogeneous cancer transcriptomes for diagnostic and therapeutic implications.

### F-circRNA: a new sight in hematologic malignancies

Chromosomal translocations give rise to oncogenic fusion gene thus encode fusion proteins which have been shown to contribute to tumorigenesis [79, 80]. PML/RARa and BCR-ABL are the most famous and recurrent fusion gene in patients with APL (acute promyelocytic leukemia) and CML (chronic granulocytic leukemia) respectively [81]. These two well-established fusion gene has become the effective therapeutic targets and reliable prognosis assessment indicators. Then whether such genomic alterations also affect noncoding RNAs, particularly circRNAs? Jlenia Guarnerio found that cancer-associated chromosomal translocations did give rise to f-circRNA (fusion circRNAs), these newly recognized cirRNAs can be generated by transcribed exons of distinct genes in translocation process [82]. Patients carrying the PML/RARa or MLL/AF9 translocation (confirmed by the use of convergent primers spanning the break point) was confirmed to display the expression of several f-circRNAs composed by different exons. Furthermore, the formation of f-circRNAs is not an exclusive feature of leukemic cells, it is also universal in tumors of different histological origins. The EWSR1/FLI1 [83] and EML4/ALK1 [84] translocation common in solid tumors such as Ewing Sarcoma and lung cancer, could also generate associated f-circRNAs. F-circRNAs promote cellular transformation, contribute cell survival upon therapy and confer resistance to treatment in tumor cells. But f-circM9 (fusion circRNA MLL/AF9) was proved probably not sufficient to trigger tumorigenesis on its own, it plays an active role in favoring leukemia progression in vivo when coupled with other oncogenic stimuli (e.g., the presence of the oncogenic fusion protein) [82]. This paper expands our current knowledge concerning molecular mechanisms of cancer onset and progression, indicating potential diagnostic and therapeutic implications.

### CircRNAs involve in signaling pathways: a potential target for treatment

There is research shows that circular and linear expression exhibits an inverse trend in many cancer related pathways. Signaling pathways like NF-kB, ILK and TGF-β, which are essential for epithelial to mesenchymal transition and typically over expressed for mRNA in metastatic tumors, are suggested down regulated for circRNA [8]. Similarly, proliferative signaling pathways (PI3k/AKT, JAK/STAT), angiogenesis pathways (VEGF, ILK) and HGF signaling were activated for linear mRNA transcripts and down regulated for circular RNA in metastases. Conversely, PTEN and PPAR signaling pathways, whose inactivation leads to carcinogenesis, were observed a downregulation in mRNA transcripts yet a corresponding upregulation of circRNA in metastases [8]. As we know, ITCH is an anti-cancer protein targeting p63, p73, and Notch1 gene and usually involve in tumor formation and chemosensitivity [85]. Cir-ITCH was shown to play an inhibitory role in the development and progression of colorectal cancer and esophageal squamous cell carcinoma and it may interact with the miRNAs (miR-7, miR-17, miR-214) thus increasing the level of ITCH, which works as inhibitor of Wnt /β-catenin signaling pathway, exerting an anti-neoplastic effect [75, 76].

### Unsolved problems in circRNA: golden mine waiting to be tapped

Majority of current studies all lend a strong support for the notion that circRNAs play fundamental roles in various disease, especially tumors. However, the function of large amounts of circRNAs which have been detected inside animal cells still remains largely unknown and awaits further detailed study. Thankfully, the results of existing researches have several interesting implications concerning circRNAs biology and toward disclosing the authentic involvement of circRNAs in various disease and cellular process.

As already stated in our previous section, the expression levels of circRNA was observed an increase after ADAR knockdown compared with the host mRNA. This finding is actually very interesting. You can imagine that if the level of ADAR decreases transiently in physiological state, it is perhaps the perfect time when circRNAs start to play a role. Prior studies have shown that stem cells can differentiate into neurons if ADAR gene expression in embryonic stem cells is reduced. Then, whether circRNA is involved in stem cell differentiation? What role dose circRNA plays in abnormal differentiation of hematopoietic stem cell associated hematological malignancy? Whether the high stability of circRNA has a relationship with the memory retention of human brain and the memory-mediated killing effect of T-lymphocytes? Maybe circRNA can help us fully understand and effectively treat the brain degenerative diseases and haematological malignancies.

We all know that circRNAs highly exist in permanent cells (neuron and cardiomyocyte) and accumulate as time goes on, then how to explain this phenomenon? Is the process of aging gives enough time for circRNAs to gather or the accumulation effect of circRNA promotes the occurrence of aging? What about the target miRNAs? Do they still bind with circRNAs sponge and increase synchronously during the aging process? What will happen to miRNAs after their release in a inactive state from the cleaved circRNAs? What contributes do the interaction between circRNA, miRNA and their parental gene serves in the process of aging and functional organ degeneration? These questions are all worthy of further study. Maybe 1 day human can unveil the mystery of aging by researching these promising RNAs.

As a footnote to distinguish varied circRNAs, the naming of circRNAs today is still based on the RNA sequencing tools, which differs significantly from each other. This chaos existing in naming method causes the difficulty of circRNA classification. A normative and widely accepted naming system is necessary. With the gradual understanding of circRNA’s biogenesis and functions, there is a hope that majority of the circRNAs can be named according to the well-studied miRNA they bind or the star gene they regulate. The most famous example is ciRS-7 and CDR1as. Considering the many-to-one relationship between miRNA, cirRNA and their mother-gene, an internal numbering system is required. With this complete naming system, we can assume a circRNA’s source and potential function after the first sight of its name, isn’t great?

## Abbreviations

ADARs: Adenosine deaminases acting on RNA
APL: Acute promyelocytic leukemia
CDK2: Cyclin-dependent kinase 2
CDR1: Cerebellum degeneration-related antigen 1
ceRNAs: Competing endogenous RNAs
CML: Chronic granulocytic leukemia
CNS: Central nervous system
CRC: Colorectal cancer
EMT: Epithelial-mesenchymal transition
EMT: Epithelial-mesenchymal transition
ESCC: Esophageal squamous cell carcinoma
GFP: Green fluorescent protein
HRCR: Heart-related circRNA
IRES: Internal ribosome entry site
LSCC: Laryngeal Squamous Cell Carcinoma
ncRNAs: Noncoding RNA
ORF: Open reading frame
ORF: Open reading frame
ORF: Infinite open reading frame
RBPs: RNA binding proteins
RCA: Rolling circle amplification
SnRNPs: Spliceosomal small nuclear ribonucleoproteins
UTR: Untranslated region
